# Selective Peroxisome Proliferator–Activated Receptor Alpha Modulators (SPPARMα): New Opportunities to Reduce Residual Cardiovascular Risk in Chronic Kidney Disease?

**DOI:** 10.1007/s11883-020-00860-w

**Published:** 2020-07-15

**Authors:** Jean-Charles Fruchart, Michel P. Hermans, Jamila Fruchart-Najib

**Affiliations:** 1 Residual Risk Reduction Initiative (R3i) Foundation, Picassoplatz 8, 4010 Basel, Switzerland; 2 Division of Endocrinology and Nutrition, Cliniques Universitaires St-Luc and Institut de Recherche Expérimentale et Clinique (IREC), Université Catholique de Louvain, Brussels, Belgium

**Keywords:** Selective peroxisome proliferator–activated receptor alpha modulator, Pemafibrate, Atherogenic dyslipidemia, Triglycerides, Chronic kidney disease, Residual cardiovascular risk

## Abstract

**Purpose of Review:**

Chronic kidney disease (CKD) poses a major global challenge, which is exacerbated by aging populations and the pandemic of type 2 diabetes mellitus. Much of the escalating burden of CKD is due to cardiovascular complications. Current treatment guidelines for dyslipidemia in CKD prioritize low-density lipoprotein cholesterol management, but still leave a high residual cardiovascular risk. Targeting elevated triglycerides and low plasma high-density lipoprotein cholesterol, a common feature of CKD, could offer additional benefit. There are, however, safety issues with current fibrates (peroxisome proliferator–activated receptor alpha [PPARα] agonists), notably the propensity for elevation in serum creatinine, indicating the need for new approaches.

**Recent Findings:**

Interactions between the ligand and PPARα receptor influence the specificity and potency of receptor binding, and downstream gene and physiological effects. The peroxisome proliferator–activated receptor alpha modulator (SPPARMα) concept aims to modulate the ligand structure so as to enhance binding at the PPARα receptor, thereby improving the ligand’s selectivity, potency, and safety profile. This concept has led to the development of pemafibrate, a novel SPPARMα agent. This review discusses evidence that differentiates pemafibrate from current fibrates, especially the lack of evidence for elevation in serum creatinine or worsening of renal function in high-risk patients, including those with CKD.

**Summary:**

Differentiation of pemafibrate from current fibrates aims to address unmet clinical needs in CKD. The ongoing PROMINENT study will provide critical information regarding the long-term efficacy and safety of pemafibrate in patients with type 2 diabetes mellitus, including those with CKD, and whether the favorable lipid-modifying profile translates to reduction in residual cardiovascular risk.

## Introduction

Diabetes and chronic kidney disease (CKD) pose major societal burdens, largely due to the associated high risk for atherosclerotic cardiovascular disease (ASCVD) [1]. The diabetes pandemic is already well recognized, with nearly half a billion individuals already affected, the vast majority with type 2 diabetes mellitus (T2DM), which is largely lifestyle-related [2]. As diabetes prevalence increases, the associated global economic burden is predicted to double by 2030, increasing from US$1.3 trillion in 2015 to US$2.2 trillion [3], with emerging economic regions facing a disproportionate increase beyond that expected based on changes in population demographics [4]. Although cardiovascular complications are a major contributor to this burden [5], the cost of care of microvascular complications, such as diabetic kidney disease, is increasingly relevant as patients survive their initial cardiovascular event as a result of therapeutic advances.

Until recently, the challenge posed by CKD, defined conventionally as estimated glomerular filtration rate (eGFR) < 60 ml/min/1.73 m^2^ or the presence of other markers of renal deterioration (including albuminuria or proteinuria) [6], has received less attention. This has gained new impetus, however, as CKD now affects 11–15% of the global population, almost doubling in some regions over the last 30 years [4, 7]. In part, the rise in CKD prevalence can be explained by population demographics, conflated by trends in T2DM, obesity, and hypertension. Declining renal function with age confers an increased cardiovascular risk, which although accelerated by comorbid conditions such as hyperglycemia and hypertension, is independent of conventional risk factors [8]. Cardiovascular complications pose the major problem, as patients with CKD are more likely to die of ASCVD than develop end-stage renal disease and require renal replacement therapy [9]. The economic burden posed by CKD is therefore substantial, especially as early-stage CKD may affect up to 35% of those aged over 70 years [10]. Not surprisingly, comorbid T2DM and CKD exacerbate this burden [11].

As evident in T2DM, dysregulation of lipid metabolism in CKD (without nephrotic syndrome) results in a dyslipidemia characterized by low plasma levels of high-density lipoprotein cholesterol (HDL-C) and elevated triglycerides (TG), usually with normal levels of low-density lipoprotein cholesterol (LDL-C) [12]. Elevated TG in this dyslipidemic profile is a surrogate for increases in TG-rich lipoproteins (very low-density lipoproteins, chylomicrons, and their remnants). This hypertriglyceridemia predominantly results from delayed catabolism due to decreased activity of lipases involved in TG lipoprotein metabolism and increased activity of lipoprotein lipase inhibitors such as apolipoprotein C-III, and to a lesser extent, increased hepatic production of TG-rich lipoproteins. The latter is especially noted in individuals with insulin resistance, such as those with T2DM. In contrast, individuals with CKD and nephrotic syndrome have a lipid profile characterized by increases in total cholesterol and LDL-C, together with hypertriglyceridemia. Dyslipidemia associated with CKD can worsen with declining kidney function due to deposition of lipids in the kidney [12, 13]. Taken together, this supports a rationale for considering dyslipidemia associated with CKD as a coronary heart disease risk similar to diabetes mellitus [1, 14].

## Unmet Therapeutic Needs in CKD

LDL-C lowering with a statin is the standard of care for management of dyslipidemia in CKD, supported by robust clinical trials that have demonstrated safety and efficacy for both lipid lowering and prevention of ASCVD events in patients with pre-end-stage renal disease [1, 6, 14, 15]. The relationship between LDL-C lowering and cardiovascular benefit demonstrated in other patient populations appears to be modified in CKD, especially as renal function worsens [16], and therefore statin treatment is not recommended in individuals with dialysis-dependent CKD with no evidence of ASCVD [1, 6]. Combination treatment with ezetimibe provides added benefit. In the Study of Heart and Renal Protection (SHARP) in 9270 patients with CKD (75% with stage 4 or stage 5 CKD at study entry), combination treatment with simvastatin and ezetimibe reduced the risk for major ASCVD events in individuals with CKD compared with placebo [17]. Added to this, post hoc analysis of the Improved Reduction of Outcomes: Vytorin Efficacy International Trial (IMPROVE-IT) showed that the combination of simvastatin-ezetimibe was more effective than simvastatin alone in individuals with moderately reduced renal function (eGFR ≤ 60 ml/min/1.73 m^2^). Relative risk reductions for the primary endpoint (a composite of cardiovascular death, major coronary event, or nonfatal stroke) were 12% and 13% for individuals with an eGFR of 60 and 45 ml/min/1.73 m^2^, respectively, compared with 9% in those with an eGFR of 75 ml/min/1.73 m^2^ [18] The number needed to treat (NNT) for ezetimibe-simvastatin treatment among individuals with the greatest renal impairment (eGFR < 45 ml/min/1.73 m2) was 12; this compares with a NNT of 24 for atorvastatin 80 mg/day in secondary prevention patients with CKD in the Treating to New Targets (TNT) study [18, 19].

Despite LDL-C lowering therapy, however, CKD patients continue to be at high residual cardiovascular risk [16], suggesting the need to consider other potential targets. One such candidate is elevated TG, a key component of the atherogenic dyslipidemia commonly seen in CKD and T2DM, supported by a growing body of evidence supporting elevated TG-rich lipoproteins and remnant cholesterol as causal for ASCVD, independent of LDL-C lowering [20–22].

Current treatment options for lowering TG in the general population, as well as in high-risk individuals with T2DM, with or without ASCVD, include high-dose icosapent ethyl (ethyl eicosapentaenoic acid, an omega-3 fatty acid) and fibrates (peroxisome proliferator–activated receptor alpha [PPARα] agonists) [23]. High-dose icosapent ethyl significantly reduced major cardiovascular events by 25% in the Reduction of Cardiovascular Events with Icosapent Ethyl-Intervention Trial (REDUCE-IT) in statin-treated T2DM patients with persistently high TG [24•]. Subsequent analyses showed that the extent of TG lowering with this therapy did not, however, equate with the expected cardiovascular event reduction, implying that effects beyond TG lowering were likely to have contributed to this cardiovascular benefit [25, 26•]. Additionally, there is no evidence from REDUCE-IT that lowering TG with icosapent ethyl reduces cardiovascular events in patients with CKD [24•]. There also may be safety issues, given increases in atrial fibrillation and serious bleeding seen with icosapent ethyl in this trial, which may relate to non-lipid effects of therapy [24•].

Current fibrates such as fenofibrate undergo renal excretion, and therefore their use is problematic in patients with CKD. Elevation in serum creatinine is well recognized with fenofibrate, which although reversible on discontinuation of treatment in patients with normal renal function [27, 28], may pose a risk for further deterioration in those with renal impairment. The burden of this is not unsubstantial, as shown in a population-level cohort study in about 80,000 patients, in which new fibrate use in older patients (aged > 65 years) increased hospitalization and nephrologist consultation due to elevation in serum creatinine [29]. Although data from the Action to Control Cardiovascular Risk in Diabetes (ACCORD) Lipid study suggested that elevation in serum creatinine with fenofibrate treatment did not predispose to renal tubular injury [30•], over long-term follow-up there was an increased risk of the major kidney outcome (a composite of incident macroalbuminuria, doubling of creatinine, self-reported need for dialysis, or death from any cause), which was driven entirely by creatinine doubling [31]. Whether this is an appropriate surrogate for progression to more severe CKD may be debated [32]; however, there is no evidence to indicate that fenofibrate treatment may mitigate renal harm in T2DM patients [31]. In addition, drug interactions with the combination of a fibrate and a statin may increase the potential for muscle problems and promote discontinuation of treatment [33]. Consequently, fibrates are not routinely recommended in individuals with pre-existing renal impairment [33, 34].

Furthermore, there is a lack of definitive evidence that fibrate treatment reduces residual cardiovascular risk. Gemfibrozil as monotherapy reduced cardiovascular events in both primary prevention and secondary prevention settings but is not recommended as add-on to a statin due to the increased risk of myopathy [35, 36]. Outcomes studies with bezafibrate or fenofibrate have been neutral [37–39], although a post hoc analysis of the major fibrate trials did, however, suggest benefit in high-risk individuals with atherogenic dyslipidemia (including those on statin treatment in the ACCORD Lipid study), when compared with those without this lipid profile [40]. In the setting of CKD there is a paucity of information. A meta-analysis suggested that fibrate treatment (predominantly as monotherapy) reduced the risk of cardiovascular events in patients with CKD, of similar magnitude to that seen in individuals with normal kidney function, although data for patients with mild to moderate CKD were limited [41]. In the ACCORD Lipid trial, 35% of patients had mild to moderate CKD. Here, the addition of fenofibrate therapy had no effect on the risk for cardiovascular events compared with statin alone, whereas in patients without CKD there was a reduction in cardiovascular death [42].

In summary, it is evident that there is an unmet need for further therapeutic approaches targeting dyslipidemia in CKD patients with or without T2DM. Could the deployment of precision medicine, targeting the PPARα receptor, overcome the safety issues recognized with fibrates?

## The SPPARMα Concept: Aiming to Improve the Benefit–Risk Profile

PPARα, a member of the PPAR family of nuclear receptors, has a critical role in the transcriptional regulation of lipoprotein metabolism, influencing the production and catabolism of TG-rich lipoproteins, HDL synthesis, as well as the β-oxidation pathway [43]. Beyond these actions, PPARα activation may also regulate glucose homeostasis, inhibit inflammation and thrombogenesis, and improve vascular function [44–46]. These activities have been extensively reviewed [47•].

The mechanism of interaction of a ligand at the PPARα receptor has been established [43]. Briefly, binding of a ligand (either a drug such as a fibrate or endogenous ligand such as prostaglandins, leukotrienes, and medium-long-chain free fatty acids), to the ligand-binding domain of the PPARα receptor induces a conformational change, which in turn facilitates recruitment of a specific profile of binding cofactors which either promote or repress expression of target genes involved in key metabolic pathways. The ligand-activated PPARα forms a heterodimeric complex with another ligand-activated nuclear receptor, the Retinoid X Receptor, which then binds to a specific DNA sequence in the promoter region of target genes. Transactivation mediated by a coactivator-acetyl transferase complex results in the expression of key genes involved in lipid metabolism. Alternatively, binding to a repressor protein prevents transcription of other genes (transrepression). PPARα ligands may share cofactors leading to the same biological response, or there may be differences in the profile of cofactors resulting in differing responses. Thus, the unique receptor–cofactor-binding profile of the PPARα ligand influences the specificity and potency of receptor binding, and downstream gene and physiological effects [47•].

Modifying the binding interactions between the ligand and the PPARα receptor therefore offers the opportunity to modulate the receptor–cofactor binding profile, and thus improve the selectivity, potency, and safety profile of the ligand compared with fibrates. This in turn would improve the benefit versus risk balance. This rationale, which has already been used successfully in the development of selective estrogen receptor modulators for breast cancer [48], underlies the SPPARMα concept [49, 50•].

Early studies investigated several potential candidates [51]. While some showed higher potency than fenofibrate in vitro, this did not translate to improved TG-lowering efficacy in patients with atherogenic dyslipidemia and/or there were safety issues [51, 52]. Deployment of a precision medicine strategy involving the design, synthesis, and rigorous screening of more than 1500 compounds identified several potential SPPARMα candidates. Of these, one—K-877, subsequently named pemafibrate—showed potent PPARα activity and selectivity, and is now licensed in Japan.

## How Does This SPPARMα Differ from Fibrates?

**Fig. 1:**
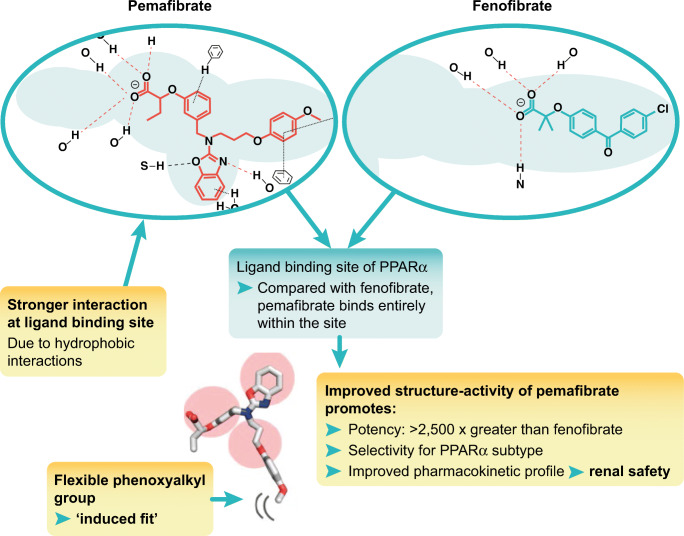
Structural differences between pemafibrate and current fibrates. Pemafibrate differs from current fibrates such as fenofibrate by the introduction of benzoxazole and phenoxyalkyl sidechains. The resulting Y-shaped structure of pemafibrate binds more strongly and entirely within the ligand-binding site, due to enhanced hydrophobic interactions. The flexibility of the phenoxyalkyl group of pemafibrate is critical in conferring an “induced-fit” with the peroxisome proliferator–activated receptor alpha (PPARα) receptor, resulting in coactivator-dependent activation as a selective PPARα modulator (SPPARMα)

Critical to differences between pemafibrate and current fibrates is the binding of each ligand within the large lipid-binding pocket of the PPARα receptor. Fibrates such as fenofibrate have a linear structure which binds to only one arm of the Y-shaped ligand-binding pocket of the PPARα receptor, thereby limiting interactions between the ligand and cofactors. Pemafibrate was synthesized by introducing benzoxazole and phenoxyalkyl sidechains, while maintaining the acidic region in its structure as in fibrates, resulting in a Y-shaped structure [53]. Because of this shape, pemafibrate binds more strongly and entirely within the lipid-binding pocket [54•] and induces different conformational changes in PPARα. The flexibility of the phenoxyalkyl group is critical in conferring this stronger “induced-fit” with the PPARα receptor, resulting in coactivator-dependent activation as a SPPARMα (Fig. 1) [55].

These structure-activity differences translated to marked increases in potency (> 2500-fold) and higher subtype selectivity for PPARα with pemafibrate compared with fenofibric acid (the active moiety of fenofibrate) [56]. Transcriptome analysis confirmed differences between these ligands, with induction of key genes regulating lipid metabolism, such as those encoding the very low-density lipoprotein receptor (*VLDLR*) and the adenosine triphosphate binding cassette transporter 1 (*ABCA1*)*,* at 10-fold lower concentration of pemafibrate than fenofibrate [57]. In vivo experimental models showed superior lipid-modifying activity with pemafibrate compared with fenofibrate, in terms of lowering TG and raising HDL-C, as well as anti-inflammatory effects [56, 58]. Together, these actions contributed to attenuation of atherosclerotic lesion development [59].

Aside from effects in metabolically active tissues, PPARα is also expressed in glomerular and renal tubular cells, where it regulates renal lipid accumulation [60]. Interestingly, in an experimental model of diabetic nephropathy (db/db mice) [61], administration of pemafibrate ameliorated this nephropathy by inhibiting deposition of lipids in the renal tubules and reducing oxidative stress. Of the mechanisms proposed to explain this effect, modulation of the renal 5′-AMP-activated protein kinase-acetyl-CoA carboxylase pathway, leading to acceleration of fatty acid β-oxidation and inhibition of fatty acid synthesis, and thus inhibition of the diacylglycerol protein kinase C NAD(P)H oxidase, may be critical [61].

Preclinical findings therefore support the SPPARMα concept. In clinical pharmacokinetic studies, pemafibrate showed a favorable profile. Unlike current fibrates, pemafibrate is not eliminated via the kidneys but instead undergoes hepatic excretion [62]. Drug-interaction studies involving coadministration of pemafibrate with six available statins also showed no clinically relevant increase in exposure to the statin [63]. Moreover, single-dose or repeated-dose studies in individuals with renal impairment showed no increase in plasma pemafibrate concentrations [62, 64•]. The key question, however, is whether these differences in potency, selectivity, and pharmacokinetics/pharmacodynamics translate to an improved benefit versus risk profile for pemafibrate versus current fibrates, especially in the context of renal safety.

## Clinical Experience with Pemafibrate

Results from clinical trials are supportive of the SPPARMα concept, and delineate differences, notably in safety, between pemafibrate and current fibrates.

### Efficacy

In a pivotal placebo-controlled phase 2 study in Japanese subjects, pemafibrate 0.2–0.4 mg daily significantly lowered remnant cholesterol and TG (by up to 80% and ~ 50%, respectively), increased HDL-C (by ~ 20%), and resulted in qualitative improvements in the atherogenic lipoprotein profile, notably reducing the proportion of small and very small LDL particles. The decrease in TG was significantly greater than with low dose fenofibrate [65].

Subsequent findings corroborated the TG-lowering efficacy of pemafibrate [66••]. In a pooled analysis of 12-week data from six placebo-controlled trials (*n* = 1253, with and without statin treatment), pemafibrate 0.4 mg daily significantly lowered TG by ~ 50% in both groups compared with placebo [67•]. There was very low interpatient variability in response, as only 1.4% in the “with-statin” group and 2.3% in the “without-statin” group did not show a decrease in TG. Another meta-analysis of seven randomized controlled trials (*n* = 1623) showed similar findings, with significant TG lowering compared with placebo (*p* < 0.001), albeit similar with fenofibrate. HDL-C, non-HDL-C levels, and the homeostasis model assessment of insulin resistance also improved with pemafibrate but not with placebo [68•]. Furthermore, data from the PROVIDE study in T2DM patients with hypertriglyceridemia showed durable responses in terms of decreases in TG and non-HDL-C and elevation in HDL-C, which were sustained over 52 weeks [69].

### Safety

Safety analyses highlight an improved benefit–risk profile for pemafibrate compared with fenofibrate. Both pooled analyses described above, showed that treatment with pemafibrate was associated with a lower incidence of adverse events compared with fenofibrate [67•, 68•]. The incidence of adverse events during 12 weeks of treatment in patients with renal impairment and concomitant statin therapy was similar in pemafibrate and placebo groups [67•]. Findings from a meta-analysis of seven studies were similar, demonstrating a significantly lower incidence of adverse events compared with fenofibrate (odds ratio 0.60; 95% confidence interval 0.49–0.73; *p* < 0.001), in particular lower frequencies of increases in hepatobiliary enzyme activity. Both alanine aminotransferase (ALT) and gamma-glutamyl transferase (GGT) activity were significantly decreased with pemafibrate treatment, compared with either fenofibrate or placebo. Importantly, the incidence of creatinine elevation was low and similar in the pemafibrate and placebo groups, and significantly less than that seen with fenofibrate treatment [68•].

### Patients with Renal Dysfunction

**Fig. 2:**
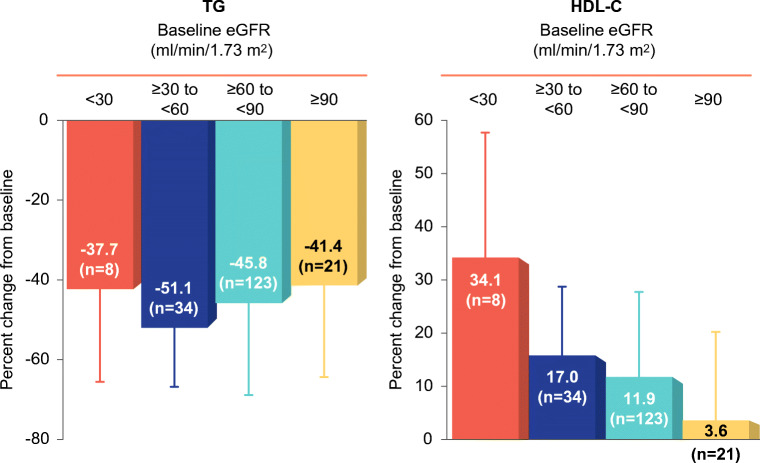
Pemafibrate in patients with impaired renal function. Treatment with pemafibrate (0.2–0.4 mg daily) led to substantial lowering of triglycerides (TG) and elevation of high-density lipoprotein cholesterol (HDL-C), with similar responses in patients with normal or impaired renal function. Data from Yokote et al. [64•]. eGFR estimated glomerular filtration rate

**Fig. 3:**
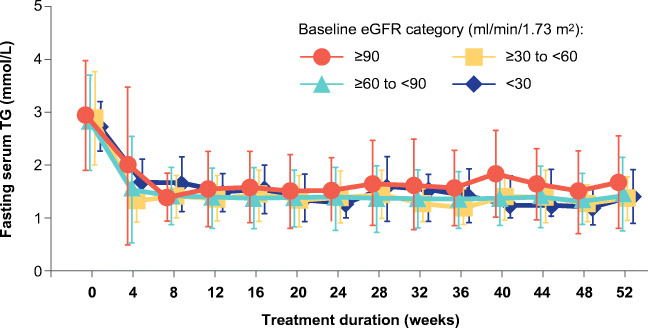
Durable lowering of triglycerides with pemafibrate. The triglyceride (TG)-lowering effects of pemafibrate (0.2–0.4 mg daily) in patients with normal or impaired renal function were sustained for 52 weeks. Reproduced with permission from Yokote et al. [64•] under Creative Commons License 4.0 (https://creativecommons.org/licenses/by/4.0/). eGFR estimated glomerular filtration rate

In pooled analyses of randomized controlled trials, concomitant renal dysfunction did not affect the efficacy or tolerability of pemafibrate-statin combination therapy [67•]. A separate study specifically evaluated the efficacy and safety of pemafibrate in dyslipidemic patients with a broad range of renal dysfunction, based on eGFR (ranging from normal, i.e., > 90 mL/min/1.73 m^2^ to < 30 mL/min/1.73 m^2^) [64•]. Pemafibrate 0.2–0.4 mg daily was administered for up to 52 weeks. At the end of the study, TG levels were reduced by 45.9% in patients receiving 0.2 mg daily, with a further decrease to 58.5% in 17 patients uptitrated to 0.4 mg daily. There was no difference in efficacy between patients with normal renal function or with renal impairment (Fig. 2). Even among patients on hemodialysis, decreases in TG ranged from 43.4 to 57.5% over 52 weeks. Patients with lower baseline eGFR exhibited the largest decreases in TG-rich lipoproteins and small LDL-C levels, as well as increases in HDL-C [64•]. The TG-lowering effects of pemafibrate were durable and sustained over 52 weeks in all patients (Fig. 3) [64•].

**Fig. 4:**
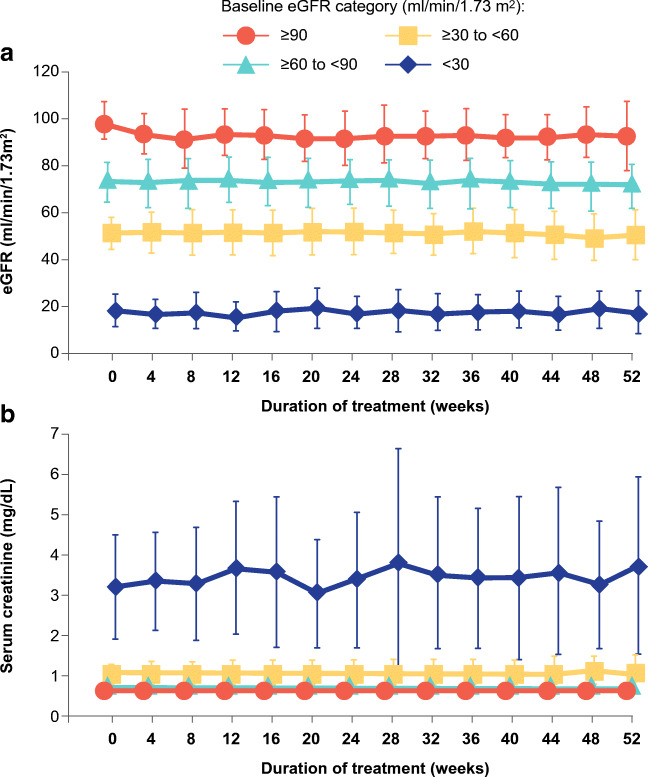
Renal safety of pemafibrate. Estimated glomerular filtration rate (eGFR) (a) and serum creatinine (b) over 52 weeks showed favorable renal safety in patients with normal and impaired renal function, treated with pemafibrate (0.2–0.4 mg daily). Reproduced with permission from Yokote et al. [64•] under Creative Commons License 4.0 (https://creativecommons.org/licenses/by/4.0/)

Importantly, baseline eGFR did not increase the incidence of adverse events [64•]. Elevation in serum creatinine (> 2 × baseline value) was not reported for any patient treated with pemafibrate, irrespective of baseline renal function. While there was a suggestion of increasing serum creatinine in patients with normal or only mild renal dysfunction (eGFR ≥ 90 mL/min/1.73 m^2^; or > 60 and < 90 mL/min/1.73 m^2^) over 52 weeks (Fig. 4) [64•], the lack of a placebo control group does not permit interpretation of the clinical context of these findings. It is, however, pertinent that the magnitude of these increases was similar to that reported in previous studies and lower than observed with fenofibrate [70–73]. Moreover, eGFR did not worsen in patients with moderate or severe renal impairment (Fig. 4). In addition, GGT and alkaline phosphatase levels decreased in all patients except those with eGFR < 30 mL/min/1.73 m^2^ over the 52-week study [64•]. A recent case-report also reported that treatment with pemafibrate 0.1 mg daily decreased excretion of urinary protein without changing eGFR or blood pressure in patients with IgA nephropathy and hypertriglyceridemia [74].

The clinical safety profile of pemafibrate in dyslipidemic patients, including those with renal dysfunction, appears favorable over 52 weeks, with no evidence to suggest risk of renal deterioration or elevation in serum creatinine. Thus, pemafibrate offers an improved renal safety profile compared with current fibrates such as fenofibrate, a distinct advantage in the setting of diabetic renal disease. It should, however, be borne in mind that these data relate to a relatively short treatment duration in clinical trials and that further long-term data in real-world clinical practice are needed to fully evaluate the safety of pemafibrate in patients with and without CKD.

## Potential for Management of Dyslipidemia in CKD

From available evidence, pemafibrate appears to have a favorable benefit–risk profile in the management of atherogenic dyslipidemia and hypertriglyceridemia. In particular, renal and hepatic safety was good, with no elevation in serum creatinine, a key issue with current fibrates such as fenofibrate, especially in patients with CKD. There was also no evidence to suggest any worsening of renal function in patients with pre-existing renal impairment as in diabetic kidney disease. This profile suggests that pemafibrate may fulfill an important unmet clinical need in this patient group.

Whether pemafibrate reduces cardiovascular events in patients with CKD is not known. This gap in evidence is being addressed by the PROMINENT study [75••], in high-risk T2DM patients (one-third without clinical ASCVD) with atherogenic dyslipidemia, defined as TG 200–499 mg/dL (2.26–5.64 mmol/L) and low HDL-C ≤ 40 mg/dl (1.03 mmol/L). Additional inclusion criteria include moderate-to-high intensity statin therapy or specified LDL-C criteria. Patients are randomized to treatment with pemafibrate 0.2 mg twice daily or placebo, and the primary outcome is a composite of myocardial infarction, ischemic stroke, hospitalization for unstable angina requiring unplanned coronary revascularization, and cardiovascular death. As mild to moderate renal dysfunction is not an exclusion criterion, PROMINENT will offer opportunities to investigate whether the addition of pemafibrate in T2DM patients with CKD reduces the residual risk of cardiovascular events in these very high-risk patients. Enrolment of the required 10,000 patients is now completed and results are anticipated in the next 2 years.

## Conclusions

CKD is a major global challenge, largely driven by escalation in the diabetes pandemic. CKD not only confers a risk of progression of renal disease, but is also associated with accelerated ASCVD, the major cause of death. As for T2DM, atherogenic dyslipidemia, the combination of elevated TG and low plasma levels of HDL-C, is a common feature. Despite guideline recommended LDL-C lowering therapy, patients with CKD remain at high residual cardiovascular risk. Current therapeutic options for the management of hypertriglyceridemia associated with CKD have issues, particularly with respect to safety.

Pemafibrate, a novel SPPARMα, may address this important unmet clinical need. Evidence to date supports a favorable benefit–risk profile for pemafibrate in the management of dyslipidemia, including among patients with CKD, with no elevation in serum creatinine, a well-recognized problem with current fibrates. The PROMINENT study will provide critical information regarding the long-term efficacy and safety of pemafibrate in T2DM, and whether its favorable lipid-modifying profile translates to reduction in residual cardiovascular risk, especially among TDM patients with CKD.

## Data Availability

Data used during the current review are available from the corresponding author on reasonable request.

## References

[1] Piepoli MF, Hoes AW, Agewall S, Albus C, Brotons C, Catapano AL, Cooney MT, Corrà U, Cosyns B, Deaton C, Graham I, Hall MS, Hobbs FDR, Løchen ML, Löllgen H, Marques-Vidal P, Perk J, Prescott E, Redon J, Richter DJ, Sattar N, Smulders Y, Tiberi M, van der Worp HB, van Dis I, Verschuren WMM, Binno S, ESC Scientific Document Group (2016). 2016 European guidelines on cardiovascular disease prevention in clinical practice: the sixth joint task force of the European Society of Cardiology and Other Societies on cardiovascular disease prevention in clinical practice (constituted by representatives of 10 societies and by invited experts) developed with the special contribution of the European Association for Cardiovascular Prevention & rehabilitation (EACPR). Eur Heart J.

[2] International Diabetes Federation. Diabetes Facts and Figures. https://www.idf.org/aboutdiabetes/what-is-diabetes/facts-figures.html (Accessed 10 February 2020).

[3] Bommer C, Sagalova V, Heesemann E, Manne-Goehler J, Atun R, Bärnighausen T, Davies J, Vollmer S (2018). Global economic burden of diabetes in adults: projections from 2015 to 2030. Diabetes Care.

[4] GBD 2015 Eastern Mediterranean Region Diabetes and Chronic Kidney Disease Collaborators. Diabetes mellitus and chronic kidney disease in the Eastern Mediterranean Region: findings from the Global Burden of Disease 2015 study. Int J Public Health. 2018;63(Suppl 1):S177–86. 10.1007/s00038-017-1014-1 PMC5973961 28776240

[5] Einarson TR, Acs A, Ludwig C, Panton UH (2018). Economic burden of cardiovascular disease in type 2 diabetes: a systematic review. Value Health.

[6] Tonelli M, Wanner C (2014). Kidney disease: improving global outcomes lipid guideline development work group members. lipid management in chronic kidney disease: synopsis of the kidney disease: improving global outcomes 2013 clinical practice guideline. Ann Intern Med.

[7] Hill NR, Fatoba ST, Oke JL, Hirst JA, O’Callaghan CA, Lasserson DS, Hobbs FDR (2016). Global prevalence of chronic kidney disease – a systematic review and meta-analysis. PLoS One.

[8] Lv JC, Zhang LX (2019). Prevalence and disease burden of chronic kidney disease. Adv Exp Med Biol.

[9] Gargiulo R, Suhail F, Lerma EV (2015). Cardiovascular disease and chronic kidney disease. Dis Mon.

[10] O'Callaghan CA, Shine B, Lasserson DS (2011). Chronic kidney disease: a large-scale population-based study of the effects of introducing the CKD-EPI formula for eGFR reporting. BMJ Open.

[11] McQueen RB, Farahbakhshian S, Bell KF, Nair KV, Saseen JJ (2017). Economic burden of comorbid chronic kidney disease and diabetes. J Med Econ.

[12] Hager MR, Narla AD, Tannock LR (2017). Dyslipidemia in patients with chronic kidney disease. Rev Endocr Metab Disord.

[13] Moorhead JF, Chan MK, El-Nahas M (1982). Lipid nephrotoxicity in chronic progressive glomerular and tubulo-interstitial disease. Lancet.

[14] Mach F, Baigent C, Catapano AL, Koskinas KC, Casula M, Badimon L, Chapman MJ, De Backer GG, Delgado V, Ference BA, Graham IM, Halliday A, Landmesser U, Mihaylova B, Pedersen TR, Riccardi G, Richter DJ, Sabatine MS, Taskinen MR, Tokgozoglu L, Wiklund O, ESC Scientific Document Group (2020). 2019 ESC/EAS Guidelines for the management of dyslipidaemias: lipid modification to reduce cardiovascular risk. Eur Heart J.

[15] Cosentino F, Grant PJ, Aboyans V, Bailey CJ, Ceriello A, Delgado V, Federici M, Filippatos G, Grobbee DE, Hansen TB, Huikuri HV, Johansson I, Jüni P, Lettino M, Marx N, Mellbin LG, Östgren CJ, Rocca B, Roffi M, Sattar N, Seferović PM, Sousa-Uva M, Valensi P, Wheeler DC, ESC Scientific Document Group (2020). 2019 ESC Guidelines on diabetes, pre-diabetes, and cardiovascular diseases developed in collaboration with the EASD. Eur Heart J.

[16] Herrington WG, Emberson J, Mihaylova B, Blackwell L, Reith C, Solbu MD, Mark PB, Fellström B, Jardine AG, Wanner C, Holdaas H, Fulcher J, Haynes R, Landray MJ, Keech A, Simes J, Collins R, Baigent C, Cholesterol Treatment Trialists' (CTT) Collaboration (2016). Impact of renal function on the effects of LDL cholesterol lowering with statin-based regimens: a meta-analysis of individual participant data from 28 randomised trials. Lancet Diabetes Endocrinol.

[17] Baigent C, Landray MJ, Reith C, Emberson J, Wheeler DC, Tomson C, Wanner C, Krane V, Cass A, Craig J, Neal B, Jiang L, Hooi LS, Levin A, Agodoa L, Gaziano M, Kasiske B, Walker R, Massy ZA, Feldt-Rasmussen B, Krairittichai U, Ophascharoensuk V, Fellstrom B, Holdaas H, Tesar V, Wiecek A, Grobbee D, de Zeeuw D, Gronhagen-Riska C, Dasgupta T, Lewis D, Herrington W, Mafham M, Majoni W, Wallendszus K, Grimm R, Pedersen T, Tobert J, Armitage J, Baxter A, Bray C, Chen Y, Chen Z, Hill M, Knott C, Parish S, Simpson D, Sleight P, Young A, Collins R, SHARP Investigators (2011). The effects of lowering LDL cholesterol with simvastatin plus ezetimibe in patients with chronic kidney disease (Study of Heart and Renal Protection): a randomised placebo-controlled trial. Lancet.

[18] Stanifer JW, Charytan DM, White J, Lokhnygina Y, Cannon CP, Roe MT, Blazing MA (2017). Benefit of ezetimibe added to simvastatin in reduced kidney function. J Am Soc Nephrol.

[19] Shepherd J, Kastelein JJ, Bittner V, Deedwania P, Breazna A, Dobson S, Wilson DJ, Zuckerman A, Wenger NK, TNT (treating to new targets) investigators (2008). Intensive lipid lowering with atorvastatin in patients with coronary heart disease and chronic kidney disease: the TNT (Treating to New Targets) study. J Am Coll Cardiol.

[20] Brunzell JD, Davidson M, Furberg CD, Goldberg RB, Howard BV, Stein JH, Witztum JL (2008). Lipoprotein management in patients with cardiometabolic risk: consensus statement from the American Diabetes Association and the American College of Cardiology Foundation. J Am Coll Cardiol.

[21] Miller M, Stone NJ, Ballantyne C, Bittner V, Criqui MH, Ginsberg HN, Goldberg AC, Howard WJ, Jacobson MS, Kris-Etherton PM, Lennie TA, Levi M, Mazzone T, Pennathur S, American Heart Association Clinical Lipidology, Thrombosis, and Prevention Committee of the Council on Nutrition, Physical Activity, and Metabolism; Council on Arteriosclerosis, Thrombosis and Vascular Biology; Council on Cardiovascular Nursing; Council on the Kidney in Cardiovascular Disease (2011). Triglycerides and cardiovascular disease: a scientific statement from the American Heart Association. Circulation.

[22] Nordestgaard BG, Varbo A (2014). Triglycerides and cardiovascular disease. Lancet.

[23] Alexopoulos AS, Qamar A, Hutchins K, Crowley MJ, Batch BC, Guyton JR (2019). Triglycerides: emerging targets in diabetes care? Review of moderate hypertriglyceridemia in diabetes. Curr Diab Rep.

[24] Bhatt DL, Steg PG, Miller M, Brinton EA, Jacobson TA, Ketchum SB, Doyle RT, Juliano RA, Jiao L, Granowitz C, Tardif JC, Ballantyne CM (2019). REDUCE-IT Investigators. Cardiovascular risk reduction with icosapent ethyl for hypertriglyceridemia. N Engl J Med.

[25] Bhatt DL, Steg PG, Miller M, Brinton EA, Jacobson TA, Jiao L, Tardif JC, Gregson J, Pocock SJ, Ballantyne CM (2019). REDUCE-IT Investigators. Reduction in first and total ischemic events with icosapent ethyl across baseline triglyceride tertiles. J Am Coll Cardiol.

[26] • Boden WE, Bhatt DL, Toth PP, Ray KK, Chapman MJ, Lüscher TF. Profound reductions in first and total cardiovascular events with icosapent ethyl in the REDUCE-IT trial: why these results usher in a new era in dyslipidaemia therapeutics. Eur Heart J. 2019. 10.1093/eurheartj/ehz778**This editorial discussed the findings and implications of the REDUCE-IT trial.** 10.1093/eurheartj/ehz778 PMC7308541 31872245

[27] Davis TM, Ting R, Best JD, Donoghoe MW, Drury PL, Sullivan DR, Jenkins AJ, O’Connell RL, Whiting MJ, Glasziou PP, Simes RJ, Kesäniemi YA, Gebski VJ, Scott RS, Keech AC, Fenofibrate Intervention and Event Lowering in Diabetes Study investigators (2011). Effects of fenofibrate on renal function in patients with type 2 diabetes mellitus: The Fenofibrate Intervention and Event Lowering in Diabetes (FIELD) study. Diabetologia..

[28] Mychaleckyj JC, Craven T, Nayak U, Buse J, Crouse JR, Elam M, Kirchner K, Lorber D, Marcovina S, Sivitz W, Sperl-Hillen J, Bonds DE, Ginsberg HN (2012). Reversibility of fenofibrate therapy–induced renal function impairment in ACCORD type 2 diabetic participants. Diabetes Care.

[29] Zhao YY, Weir MA, Manno M, Cordy P, Gomes T, Hackam DG, Juurlink DN, Mamdani M, Moist L, Parikh CR, Paterson JM, Wald R, Yao Z, Garg AX (2012). New fibrate use and acute renal outcomes in elderly adults: a population-based study. Ann Intern Med.

[30] • Chauhan K, Nadkarni GN, Debnath N, Chan L, Saha A, Garg AX, et al. The association of fenofibrate with kidney tubular injury in a subgroup of participants in the ACCORD Trial. Clin J Am Soc Nephrol. 2019;14:1521–3 **This report from the ACCORD Lipid trial showed that treatment with fenofibrate affected renal function in patients with type 2 diabetes mellitus.** 10.2215/CJN.00370119 PMC6777587 31409596

[31] Mottl AK, Buse JB, Ismail-Beigi F, Sigal RJ, Pedley CF, Papademetriou V, Simmons DL, Katz L, Mychaleckyj JC, Craven TE (2018). Long-term effects of intensive glycemic and blood pressure control and fenofibrate use on kidney outcomes. Clin J Am Soc Nephrol.

[32] Wong MG, Heerspink HJL, Perkovic V (2018). ACCORDION: ensuring that we hear the music clearly. Clin J Am Soc Nephrol.

[33] Davidson MH, Armani A, McKenney JM, Jacobson TA (2007). Safety considerations with fibrate therapy. Am J Cardiol.

[34] Choi HD, Shin WG, Lee JY, Kang BC (2015). Safety and efficacy of fibrate-statin combination therapy compared with fibrate monotherapy in patients with dyslipidemia: a meta-analysis. Vascul Pharm.

[35] Frick MH, Elo O, Haapa K, Heinonen OP, Heinsalmi P, Helo P, Huttunen JK, Kaitaniemi P, Koskinen P, Manninen V (1987). Helsinki Heart Study: primary-prevention trial with gemfibrozil in middle-aged men with dyslipidemia. Safety of treatment, changes in risk factors, and incidence of coronary heart disease. N Engl J Med.

[36] Rubins HB, Robins SJ, Collins D, Fye CL, Anderson JW, Elam MB, Faas FH, Linares E, Schaefer EJ, Schectman G, Wilt TJ, Wittes J (1999). Gemfibrozil for the secondary prevention of coronary heart disease in men with low levels of high-density lipoprotein cholesterol. Veterans Affairs High-Density Lipoprotein Cholesterol Intervention Trial Study Group. N Engl J Med.

[37] The BIP Study Group (2000). Secondary prevention by raising HDL cholesterol and reducing triglycerides in patients with coronary artery disease. The Bezafibrate Infarction Prevention (BIP) study. Circulation..

[38] Keech A, Simes RJ, Barter P, Best J, Scott R, Taskinen MR, Forder P, Pillai A, Davis T, Glasziou P, Drury P, Kesäniemi YA, Sullivan D, Hunt D, Colman P, d'Emden M, Whiting M, Ehnholm C, Laakso M, FIELD study investigators (2005). Effects of long-term fenofibrate therapy on cardiovascular events in 9795 people with type 2 diabetes mellitus (the FIELD study): randomised controlled trial. Lancet.

[39] Ginsberg HN, Elam MB, Lovato LC, Crouse JR, Leiter LA, Linz P, Friedewald WT, Buse JB, Gerstein HC, Probstfield J, Grimm RH, Ismail-Beigi F, Bigger JT, Goff DC, Cushman WC, Simons-Morton DG, Byington RP, ACCORD Study Group (2010). Effects of combination lipid therapy in type 2 diabetes mellitus. N Eng J Med.

[40] Sacks FM, Carey VJ, Fruchart JC (2010). Combination lipid therapy in type 2 diabetes. N Engl J Med.

[41] Jun M, Zhu B, Tonelli M, Jardine MJ, Patel A, Neal B, Liyanage T, Keech A, Cass A, Perkovic V (2012). Effects of fibrates in kidney disease a systematic review and meta-analysis. J Am Coll Cardiol.

[42] Papademetriou V, Lovato L, Tsioufis C, Cushman W, Applegate WB, Mottle A, Punthakee Z, Nylen E, Doumas M, ACCORD Study Group (2017). Effects of high density lipoprotein raising therapies on cardiovascular outcomes in patients with type 2 diabetes mellitus, with or without renal impairment: the Action to Control Cardiovascular Risk in Diabetes study. Am J Nephrol.

[43] Lefebvre P, Chinetti G, Fruchart JC, Staels B (2006). Sorting out the roles of PPAR alpha in energy metabolism and vascular homeostasis. J Clin Invest.

[44] Gross B, Pawlak M, Lefebvre P, Staels B (2017). PPARs in obesity induced T2DM, dyslipidaemia and NAFLD. Nat Rev Endocrinol.

[45] Delerive P, De Bosscher K, Besnard S, Vanden Berghe W, Peters JM, Gonzalez FJ (1999). Peroxisome proliferator-activated receptor alpha negatively regulates the vascular inflammatory gene response by negative cross-talk with transcription factors NFkappaB and AP-1. J Biol Chem.

[46] Perreault L, Bergman BC, Hunerdosse DM, Howard DJ, Eckel RH (2011). Fenofibrate administration does not affect muscle triglyceride concentration or insulin sensitivity in humans. Metabolism..

[47] Bougarne N, Weyers B, Desmet SJ, Deckers J, Ray DW, Staels B, De Bosscher K (2018). Molecular actions of PPARα in lipid metabolism and inflammation. Endocr Rev.

[48] Lewis JS, Jordan VC (2005). Selective estrogen receptor modulators (SERMs): mechanisms of anticarcinogenesis and drug resistance. Mutat Res.

[49] Fruchart JC (2013). Selective peroxisome proliferator-activated receptor α modulators (SPPARMα): the next generation of peroxisome proliferator-activated receptor α-agonists. Cardiovasc Diabetol.

[50] Yamashita S, Masuda D, Matsuzawa Y (2020). Pemafibrate, a new selective PPARα modulator: drug concept and its clinical applications for dyslipidemia and metabolic diseases. Curr Atheroscler Rep.

[51] Ferri N, Corsini A, Sirtori C, Ruscica M (2017). PPAR-α agonists are still on the rise: an update on clinical and experimental findings. Expert Opin Investig Drugs.

[52] Nissen SE, Nicholls SJ, Wolski K, Howey DC, McErlean E, Wang MD, Gomez EV, Russo JM (2007). Effects of a potent and selective PPAR-alpha agonist in patients with atherogenic dyslipidemia or hypercholesterolemia: two randomized controlled trials. JAMA..

[53] Yamazaki Y, Abe K, Toma T, Nishikawa M, Ozawa H, Okuda A, Araki T, Oda S, Inoue K, Shibuya K, Staels B, Fruchart JC (2007). Design and synthesis of highly potent and selective human peroxisome proliferator-activated receptor alpha agonists. Bioorg Med Chem Lett.

[54] Yamamoto Y, Takei K, Arulmozhiraja S, Sladek V, Matsuo N, Han SI, Matsuzaka T, Sekiya M, Tokiwa T, Shoji M, Shigeta Y, Nakagawa Y, Tokiwa H, Shimano H (2018). Molecular association model of PPARα and its new specific and efficient ligand, pemafibrate: structural basis for SPPARMα. Biochem Biophys Res Commun.

[55] Kawasaki M, Kambe A, Yamamoto Y, Arulmozhiraja S, Ito S, Nakagawa Y, et al. Elucidation of molecular mechanism of a selective PPARα modulator, pemafibrate, through combinational approaches of X-ray crystallography, thermodynamic analysis, and first-principle calculations. Int J Mol Sci. 2020;21. 10.3390/ijms21010361. 10.3390/ijms21010361 PMC6981837 31935812

[56] Fruchart JC (2017). Pemafibrate (K-877), a novel selective peroxisome proliferator-activated receptor alpha modulator for management of atherogenic dyslipidaemia. Cardiovasc Diabetol.

[57] Raza-Iqbal S, Tanaka T, Anai M, Inagaki T, Matsumura Y, Ikeda K, Taguchi A, Gonzalez FJ, Sakai J, Kodama T (2015). Transcriptome analysis of K-877 (a novel selective PPARα modulator (SPPARMα))-regulated genes in primary human hepatocytes and the mouse liver. J Atheroscler Thromb.

[58] Sasaki Y, Raza-Iqbal S, Tanaka T, Murakami K, Anai M, Osawa T, et al. Gene expression profiles induced by a novel selective peroxisome proliferator-activated receptor α modulator (SPPARMα) pemafibrate. Int J Mol Sci. 2019;20. 10.3390/ijms20225682. 10.3390/ijms20225682 PMC6888257 31766193

[59] Hennuyer N, Duplan I, Paquet C, Vanhoutte J, Woitrain E, Touche V, Colin S, Vallez E, Lestavel S, Lefebvre P, Staels B (2016). The novel selective PPARα modulator (SPPARMα) pemafibrate improves dyslipidemia, enhances reverse cholesterol transport and decreases inflammation and atherosclerosis. Atherosclerosis.

[60] Braissant O, Foufelle F, Scotto C, Dauça M, Wahli W (1996). Differential expression of peroxisome proliferator-activated receptors (PPARs): tissue distribution of PPAR-alpha, −beta, and -gamma in the adult rat. Endocrinology..

[61] Maki T, Maeda Y, Sonoda N, Makimura H, Kimura S, Maeno S, Takayanagi R, Inoguchi T (2017). Renoprotective effect of a novel selective PPARα modulator K-877 in db/db mice: a role of diacylglycerol-protein kinase C-NAD(P) H oxidase pathway. Metabolism.

[62] Hounslow N, Mair S, Suganami H, Nakamura M (2018). Pemafibrate has high bioavailability and is principally excreted via the liver. Atheroscler Suppl.

[63] Hounslow N, Suganami H, Nakamura M (2018). Pemafibrate minimally affected the systemic exposure of statins, and vice versa, in healthy male volunteers. Atheroscler Suppl..

[64] • Yokote K, Yamashita S, Arai H, Araki E, Suganami H, Ishibashi S, et al. Long-term efficacy and safety of pemafibrate, a novel selective peroxisome proliferator-activated receptor-α modulator (SPPARMα), in dyslipidemic patients with renal impairment. Int J Mol Sci. 2019;20(3):706. **Excellent overview of the renal safety of pemafibrate based on clinical trial experience in Japan.** 10.3390/ijms20030706 PMC6386904 30736366

[65] Ishibashi S, Yamashita S, Arai H, Araki E, Yokote K, Suganami H, Fruchart JC, Kodama T, K-877-04 study group (2016). Effects of K-877, a novel selective PPARα modulator (SPPARMα), in dyslipidaemic patients: a randomized, double blind, active- and placebo-controlled, phase 2 trial. Atherosclerosis..

[66] Fruchart JC, Santos RD, Aguilar-Salinas C, Aikawa M, Al Rasadi K, Amarenco P, Barter PJ, Ceska R, Corsini A, Després JP, Duriez P, Eckel RH, Ezhov MV, Farnier M, Ginsberg HN, Hermans MP, Ishibashi S, Karpe F, Kodama T, Koenig W, Krempf M, Lim S, Lorenzatti AJ, McPherson R, Nuñez-Cortes JM, Nordestgaard BG, Ogawa H, Packard CJ, Plutzky J, Ponte-Negretti CI, Pradhan A, Ray KK, Reiner Ž, Ridker PM, Ruscica M, Sadikot S, Shimano H, Sritara P, Stock JK, Su TC, Susekov AV, Tartar A, Taskinen MR, Tenenbaum A, Tokgözoğlu LS, Tomlinson B, Tybjærg-Hansen A, Valensi P, Vrablík M, Wahli W, Watts GF, Yamashita S, Yokote K, Zambon A, Libby P (2019). The selective peroxisome proliferator-activated receptor alpha modulator (SPPARMα) paradigm: conceptual framework and therapeutic potential: a consensus statement from the International Atherosclerosis Society (IAS) and the Residual Risk Reduction Initiative (R3i) Foundation. Cardiovasc Diabetol.

[67] • Yamashita S, Arai H, Yokote K, Araki E, Matsushita M, Nojima T, et al. Efficacy and safety of pemafibrate, a novel selective peroxisome proliferator-activated receptor α modulator (SPPARMα): pooled analysis of phase 2 and 3 studies in dyslipidemic patients with or without statin combination. Int J Mol Sci. 2019. 10.3390/ijms20225537**Pooled efficacy and safety analysis of clinical trials with pemafibrate in Japan.** 10.3390/ijms20225537 PMC6888510 31698825

[68] Ida S, Kaneko R, Murata K (2019). Efficacy and safety of pemafibrate administration in patients with dyslipidemia: a systematic review and meta-analysis. Cardiovasc Diabetol.

[69] Araki E, Yamashita S, Arai H, Yokote K, Satoh J, Inoguchi T, Nakamura J, Maegawa H, Yoshioka N, Tanizawa Y, Watada H, Suganami H, Ishibashi S (2019). Efficacy and safety of pemafibrate in people with type 2 diabetes and elevated triglyceride levels: 52-week data from the PROVIDE study. Diabetes Obes Metab.

[70] Arai H, Yamashita S, Yokote K, Araki E, Suganami H, Ishibashi S (2017). Efficacy and safety of K-877, a novel selective peroxisome proliferator-activated receptor alpha modulator (SPPARMalpha), in combination with statin treatment: two randomised, double-blind, placebo-controlled clinical trials in patients with dyslipidaemia. Atherosclerosis..

[71] Ishibashi S, Arai H, Yokote K, Araki E, Suganami H, Yamashita S (2018). Efficacy and safety of pemafibrate (K-877), a selective peroxisome proliferator-activated receptor alpha modulator, in patients with dyslipidemia: results from a 24-week, randomized, double blind, active-controlled, phase 3 trial. J Clin Lipidol.

[72] Araki E, Yamashita S, Arai H, Yokote K, Satoh J, Inoguchi T, Nakamura J, Maegawa H, Yoshioka N, Tanizawa Y (2018). Effects of pemafibrate, a novel selective PPARalpha modulator, on lipid and glucose metabolism in patients with type 2 diabetes and hypertriglyceridemia: a randomized, double-blind, placebo-controlled, phase 3 trial. Diabetes Care.

[73] Arai H, Yamashita S, Yokote K, Araki E, Suganami H, Ishibashi S (2018). Efficacy and safety of pemafibrate versus fenofibrate in patients with high triglyceride and low HDL cholesterol levels: a multicenter, placebo-controlled, double-blind, randomized trial. J Atheroscler Thromb.

[74] Tanaka A, Nakamura T, Sato E, Chihara A, Node K (2020). Effect of pemafibrate, a novel selective peroxisome proliferator-activated receptor-alpha modulator (SPPARMα), on urinary protein excretion in IgA nephropathy with hypertriglyceridemia. CEN Case Rep.

[75] Pradhan AD, Paynter NP, Everett BM, Glynn RJ, Amarenco P, Elam M, Ginsberg H, Hiatt WR, Ishibashi S, Koenig W, Nordestgaard BG, Fruchart JC, Libby P, Ridker PM (2018). Rationale and design of the pemafibrate to reduce cardiovascular outcomes by reducing triglycerides in patients with diabetes (PROMINENT) study. Am Heart J.

